# InvBFM: finding genomic inversions from high-throughput sequence data based on feature mining

**DOI:** 10.1186/s12864-020-6585-1

**Published:** 2020-03-05

**Authors:** Zhongjia Wu, Yufeng Wu, Jingyang Gao

**Affiliations:** 1 College of Information Science and Technology, Beijing University of Chemical Technology, Beijing, People’s Republic of China; 2 Department of Computer Science and Engineering, University of Connecticut, Storrs, Connecticut USA

**Keywords:** Genomics, High-throughput sequencing, Structural variation, Inversion, Support vector machine

## Abstract

**Background:**

Genomic inversion is one type of structural variations (SVs) and is known to play an important biological role. An established problem in sequence data analysis is calling inversions from high-throughput sequence data. It is more difficult to detect inversions because they are surrounded by duplication or other types of SVs in the inversion areas. Existing inversion detection tools are mainly based on three approaches: paired-end reads, split-mapped reads, and assembly. However, existing tools suffer from unsatisfying precision or sensitivity (eg: only 50~60% sensitivity) and it needs to be improved.

**Result:**

In this paper, we present a new inversion calling method called InvBFM. InvBFM calls inversions based on feature mining. InvBFM first gathers the results of existing inversion detection tools as candidates for inversions. It then extracts features from the inversions. Finally, it calls the true inversions by a trained support vector machine (SVM) classifier.

**Conclusions:**

Our results on real sequence data from the 1000 Genomes Project show that by combining feature mining and a machine learning model, InvBFM outperforms existing tools. InvBFM is written in Python and Shell and is available for download at https://github.com/wzj1234/InvBFM.

## Background

It is widely known that genomic variation plays an important role in shaping the genetic diversity of populations. Recently, high-throughput sequencing data becomes the mature type of genomic data used in research. Finding genomic variations from high-throughput sequence data has become a major objective for large-scale genomics studies, such as the 1000 Genomics Project [1]. There are various types of genomic variations, including single nucleotide polymorphisms (SNPs), short (say 50 bp or less) deletions or insertions (indels) and SVs (which are usually longer than 50 bp). There are different types of SVs, including insertion, deletion, copy number variation, and inversion. While some types of SVs (e.g. deletion) have been very actively studied (e.g. [2–6]), other types of SVs such as inversion are less studied. Different from deletion calling where there are a growing list of deletion calling tools, there are less tools for finding inversions. We note that the impact of inversions can have large effect on an organism [7]. For example, inversion inhibits recombination in heterokaryons, which may lead to distinct gene-expression patterns. Inversion may also directly influence gene structure or regulation in different ways as well as secondary mutations in the offspring. In addition, inversion may cause diseases such as hemophilia A [8], Hunter syndrome [9] and increase the risk of infertility or miscarriage [10]. Therefore, developing effective inversion calling tools may potentially be very useful.

We focus on calling inversions from mapped sequence data (i.e. paired-end reads). Calling genomic variations from mapped sequence reads is usually based on the following three mapped sequence properties (called signatures): insert size from mapped paired-end reads (ISPE), split-mapped reads, and read depth. Note that there are also approaches performing sequence assembly. Existing inversion calling methods usually use these signatures. Pindel [11] only uses split-mapped reads. Delly [12] and Lumpy [13] are based on paired-end reads and split-mapped reads. All these three tools have been used in the 1000 Genomes Project. We note that although Delly and Lumpy use the same sets of signatures, they appear to perform differently. This implies that these tools are individually engineered in different aspects in order to call inversions more accurately. Our experience indicates that none of these tools clearly outperforms the others. A natural approach for calling inversions accurately is using machine learning: we extract various sequence features and treat inversion calling as a classification problem in machine learning. Previously, we have developed machine learning approaches for finding deletions from sequence data [14–17]. A main challenge for finding inversions from sequence data with machine learning is that inversions are relatively rare. There are not many known inversions in the benchmark data (e.g. from the 1000 Genomes Project).

In this paper, we develop a new inversion calling approach, called InvBFM. InvBFM uses multiple relevant sequence properties (called features). InvBFM mines features that are unique to both wild-type sequence and inversions, and trains a model based on these features using simulated data. Then InvBFM calls inversions based on the model with real data by examining each candidate inversion site found by multiple inversion calling results. We demonstrate that InvBFM outperforms existing inversion calling tools on real data. InvBFM in Python and Shell is available for download at https://github.com/wzj1234/InvBFM.

## Results and discussion

### Analysis of features

**Fig. 1:**
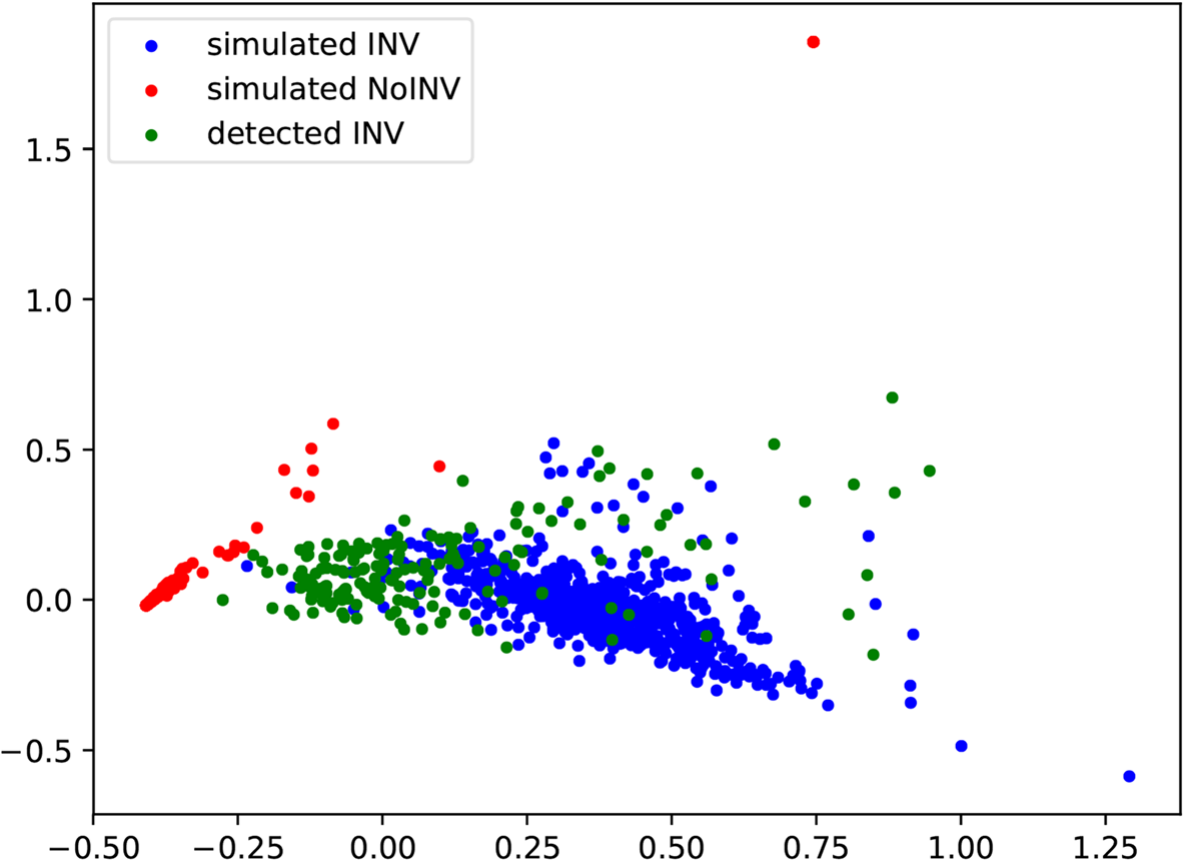
Visualization of features in simulation and benchmark. All features involved in this image are processed by PCA. The red dots mean wild-type features from simulated data, and the blue dots mean the inversions’ features from simulated data. The green dots’ features come from benchmark of inversion in real data. The green and blue dots are clustered and separated from the red dots, which indicate the features mined by InvBFM are effective

We first analyze the correlation between the numerical features and the target value. This helps to evaluate the feasibility of using the features of simulated data to train the SVM classifier and then generalize the real data features. InvBFM converts the 15-dimensional feature space which the initially extracted features of inversion are mapped into two dimensions via the principal component analysis (PCA) in order to be visualized as shown in Fig.1. In Fig.1, the blue dots represent the inversions’ features of the simulated inversions, which are extracted from simulated BAM files and then converted into 2-dimensional feature. The red dots indicate the features of wild-type in simulated data, which are also extracted from simulated data and then mapped into 2-dimensional features. The green dots correspond to the converted 2-dimensional features which are extracted from the 102 real samples near the inversion area recorded in the benchmark. It is evident that the blue and green dots, representing the inversions’ features, are well clustered. The red dots, which represent wild-type features, are clearly separated. This shows that the 15 extracted features are justifiable. That is, the features used by InvBFM are correlated well to whether the target value of the inversion occurs or not.

In order to further demonstrate that the feature selection indeed selects more effective features, we compare the detection results by choosing a different number of inversion features, as shown in Table 1. The threshold here is chosen to be three times ISPE. Features_15_ refers to the results of calling inversion for 15 features extracted initially, and Feature_8_ refers to the results of 8 features selected only by chi-square test. InvBFM uses 8 features selected in Feature_8_ and two additional features. It is obvious that the Feature_8_ has 2% improvement in precision, and about 1% improvement in recall over the original Feature_15_. Although InvBFM only leads to a small improvement in precision and recall compared to Feature_8_, it is the best of the three. Thus, feature selection in InvBFM using both chi-square test and experience indeed makes the detection of inversion more effective.

**Table 1:** Comparison of different features

Feature Version	No. Calls	TP_0_	FN	TP	FP	Precision	Recall
Features_15_	1468	168	70	478	990	32.56%	70.59%
Features_8_	1386	170	68	479	907	34.56%	71.43%
**InvBFM**	**1379**	**170**	**68**	**479**	**900**	**34.73%**	**71.43%**

Features_15_ means the first 15 features are extracted, Features_8_ means selecting 8 numeral features by the chi-square test from Features_15_, InvBFM means the union of Features_8_ and 2 features that lead to better results in practice. No.Calls: detected inversion count. *TP* true positive, *TP*_***0***_ remove repeats of TP, *FP* false positive, *FN* false negative. The kernel of SVM is linear, with the penalty factor of 0.1 and the gamma of 20

For measuring the difference in performance between selected the 8 and 10 features, we use a 10-fold cross-validation and take the average values of 100 times from all simulated data and the 204 real samples are downloaded from 1000 Genomes Project to confirm the effort of the two additional features based on experience. The dataset contains a total of 5491 breakpoints of inversion and wild-type from simulated data and real data. The comparison of classification results of the 10 features by InvBFM and the 8 features by chi-square test is shown in Table 2, and we just focus on the occurrence of inversion. In this experiment, we consider it is the true inversion and set label 1 of the breakpoints within 3 times ISPE of the benchmark, otherwise set label 0 as wild-type. Then, we extract the 8 features and the 10 features around each breakpoint respectively mentioned above. For 10-fold cross-validation, we random shuffle the whole dataset, doing 100 times of 10-fold cross-validation and evaluating the average results of the validation set. In this part, we only verify the critical SVM process. Our results here are based on the comparison of expected labels. The parameter settings of the SVM in Table 2 are the same as those in Table 1. As shown by the mean results of 100 times at 10-fold cross-validations, it is verified that the 10 features selected by InvBFM are better than the 8 features in recall and F1-score, although their precisions are very similar.

**Table 2:** Result of 10-fold cross-validation on the 8 features and 10 features

Feature Version	TP	TN	FP	FN	Precision	Recall	F1-score
Features_8_	111	366	19	51	85.38%	68.51%	76.03%
**InvBFM**	133	363	22	29	85.81%	82.10%	83.91%

Threshold = 3*ISPE. The kernel of SVM is linear, with the penalty factor of 0.1 and the gamma of 20

SVM is used by InvBFM to generalize real samples’ features. Different parameters in SVM may lead to different results. Table 3 shows the performance about precision, recall and F1-score of InvBFM in SVM with different parameters. Meanwhile, in Radial Basis Function (RBF), the kernel of SVM, penalty factor sets 8 and gamma sets 0.01 give the best result.

**Table 3:** Precision, recall and F1-score of InvBFM in SVM with different parameter

Para.	Precision	Recall	F1-score
c:32, g:5	25.51%	72.69%	37.77%
c:32, g:1	26.71%	72.27%	39.01%
c:32, g:0.1	28.32%	72.27%	40.69%
c:32, g:0.01	29.94%	72.27%	42.34%
c:8, g:0.01	32.15%	71.85%	44.42%

Threshold = 3*ISPE. The kernel of SVM is RBF, “c” stands for penalty factor and “g” stands for gamma

In order to evaluate the impact of different inversion frequencies in tools, we analyze the tools’ sensitivity in different inversion frequencies. The result is shown in Table 4. InvBFM gets the best results than existing inversion callers.

**Table 4:** Sensitivity of multiple tools on different frequencies on 102 samples of 1000 Genomes Project

INV. Freq.	INV. No.	Delly	Lumpy	LumpyEP	Pindel	InvBFM
1~10	10	85.71%	35.71%	35.71%	50.00%	89.29%
11~50	3	51.69%	1.12%	1.12%	31.46%	58.43%
> 50	2	78.51%	44.63%	41.32%	33.06%	79.34%

Threshold = 3*ISPE. The kernel of SVM is linear, with the penalty factor of 0.1 and the gamma of 20

Moreover, the sensitivity of inversion length on the detected results are shown in Table 5. Lumpy and LumpyEP are the most unstable. InvBFM also performs the best.

**Table 5:** Sensitivity of multiple tools on inversion length on 102 samples of the 1000 Genomes Project

INV. Len. (k)	INV. No.	Delly	Lumpy	LumpyEP	Pindel	InvBFM
0.3~0.5	10	80%	90%	90%	30%	90%
0.5~1	42	76.19%	0	0	47.62%	80.95%
1~2.5	73	86.30%	65.75%	60.27%	61.36%	87.67%
2.5~5	111	54.05%	6.31%	6.31%	27.93%	54.95%
5~10	2	100%	50%	50%	50%	100%

Threshold = 3*ISPE. The kernel of SVM is linear, with the penalty factor of 0.1 and the gamma of 20

### Accuracy of inversion calling

The inversion in our study is assumed to be longer than the mean ISPE of the sample. We ignore the case where the inversion length of candidate inversions is less than the sample mean ISPE. In addition, the basis for determining whether a predicted region is a true inversion is to compare the left and right breakpoints of the predicted inversion with those of the benchmark, with the threshold being 1, 2, or 3 times the average ISPE of the corresponding sequence reads. That is, if the distance between the called breakpoints is more than this threshold, we consider that the predicted inversion is not true. Experiments show that all tools perform better when the threshold is 3 times of the sample mean ISPE.

The comparison of the experimental results of different thresholds with different tools of inversion calls is shown in Table 6. The denominator value in the InvBFM row represents the union of the three tools. The numerator is a set of true inversion generalized by the SVM classifier of InvBFM, which is the value involved in the calculation. In addition, LumpyEP is the abbreviation of Lumpy express tool also released by Lumpy. Since LumpyEP results are not exactly the same as Lumpy, its results of calling inversion are also shown in Table 6. TP_0_ indicates the number of non-repetitive regions in the reference benchmark that have records and are judged to be true inversion by tools, i.e., regions of TP after removing repeat inversions. F1-score is a comprehensive evaluation indicator combining precision and recall. The relevant indicators in Table 6 are calculated by the formulas shown in eq. (1) and (2). In addition, InvBFM represents the training result of 10 features selected by the feature selection method mentioned above.

**Table 6:** Result of different tools with different threshold values (used to determine when a called inversion matches benchmark)

Thre-shold	Tool	No. Calls	TP	TP_0_	FP	FN	Precision	Recall	F1-score
ISPE	**Delly**	1142	183	150	959	88	16.02%	63.03%	25.55%
	**Lumpy**	66	51	51	15	187	77.27%	21.42%	33.55%
	**LumpyEP**	62	47	47	15	191	75.81%	19.75%	31.33%
	**Pindel**	649	84	79	565	159	12.94%	33.19%	18.62%
	**InvBFM**	$\frac{\mathbf{1379}}{\mathbf{1919}}$	$\frac{\mathbf{359}}{\mathbf{365}}$	$\frac{\mathbf{163}}{\mathbf{166}}$	**1020**	**75**	**26.03%**	**68.49%**	**37.73%**
ISPE *2	**Delly**	1142	244	164	898	74	21.37%	68.91%	32.62%
	**Lumpy**	66	65	65	1	173	98.48%	27.31%	42.76%
	**LumpyEP**	62	60	60	2	178	96.77%	25.21%	40.00%
	**Pindel**	649	99	82	550	156	15.25%	34.45%	21.14%
	**InvBFM**	$\frac{\mathbf{1379}}{\mathbf{1919}}$	$\frac{\mathbf{462}}{\mathbf{468}}$	$\frac{\mathbf{167}}{\mathbf{172}}$	**917**	**71**	**33.50%**	**70.17%**	**45.35%**
ISPE *3	**Delly**	1142	258	165	884	73	22.59%	69.33%	34.08%
	**Lumpy**	66	65	65	1	173	98.48%	27.31%	42.76%
	**LumpyEP**	62	61	61	1	177	98.39%	25.63%	40.67%
	**Pindel**	649	101	82	548	156	15.56%	34.45%	21.44%
	**InvBFM**	$\frac{\mathbf{1379}}{\mathbf{1919}}$	$\frac{\mathbf{479}}{\mathbf{485}}$	$\frac{\mathbf{170}}{\mathbf{173}}$	**900**	**68**	**34.73%**	**71.43%**	**46.74%**

No.Calls: detected inversion count. *TP* true positive, *TP*_***0***_ remove repeats of TP, *FP* false positive, *FN* false negative. The kernel of SVM is linear, with the penalty factor of 0.1 and the gamma of 20

We can see from the results that Delly’s recall achieves the best results among the three existing tools, regardless of different thresholds. Lumpy is the best in precision of the three existing tools in precision but with recall less than 30%, which is the lowest. Pindel is the worst in the precision and F1-score indicators. The recall and F1-score of InvBFM perform the best with the 10 features. InvBFM improves the F1-score by more than 10% than Delly, which achieves the highest recall among the existing tools. Therefore, InvBFM performs better in inversion calling compared with existing tools.

1 $$ FP= No. Calls- TP, FN= benchmark-{TP}_0 $$

2 $$ Precision=\frac{TP}{No. Calls}, Recall=\frac{TP_0}{benchmark},F1- score=\frac{2\ast Precision\ast Recall}{Precision+ Recall} $$

10-fold cross-validation was used to compare the performances of InvBFM and other tools for calling inversion. The dataset we used in this part is the same as the data in Table 2 from the simulated data and 204 real samples, which contains a total of 5491 inversion and wild-type breakpoints. The result of detected breakpoints of real data from all the tools are shown in Table 7. Under the threshold of 3 times ISPE, when the breakpoints deviation is within the threshold, it is considered to be a true inversion and we label it as 1; otherwise, it is set to 0. On this basis, we use Delly, Pindel, Lumpy, LumpyEP and InvBFM to call inversions and compare their results with the expected label to calculate various indicators, rather than comparing predicted inversion to the benchmark in Table 6. In each 10-fold cross-validation process, we randomly shuffled all the data, and divided the data set into 10 parts averagely. We used 9 parts for training and 1 part for validation. We repeat the process 10 times to make the validation set fully cover the whole data set. Only InvBFM involves the training process. So, the SVM classifier is trained using the inversion features of the training set for each training step. Since the validation set is used to assess the calling results of each tool, all the tools in Table 7 need to verify that each inversion is correctly detected in the validation set. The specific results of mean values in 100 times of 10-fold cross-validation are shown in Table 7. It is worth mentioning that since the InvBFM is based on modelling SVM using features from simulated data, Table 7 filters out the breakpoints in simulated data and only verifies the performance of the breakpoints from real data comparing their expected labels. The results in Table 7 verify that our InvBFM does perform optimally on the comprehensive performance of F1-score for detecting inversion without overfitting.

**Table 7:** Result of 10-fold cross-validation on the different tools

Tool	TP	TN	FP	FN	Precision	Recall	F1-score
Delly	85.30	124.10	186.70	0.90	31.36%	98.96%	47.63%
Lumpy	52.70	289.60	1.20	53.50	97.77%	49.62%	65.83%
LumpyEP	48.50	289.80	1.00	57.70	97.98%	45.67%	62.30%
Pindel	50.30	146.70	164.10	35.90	23.46%	58.35%	33.47%
**InvBFM**	**59.72**	**288.47**	**22.33**	**26.47**	**72.78**%	**69.29**%	**70.99**%

Threshold = 3*ISPE. The kernel of SVM is linear, with the penalty factor of 0.1 and the gamma of 20

## Conclusions

This paper proposes InvBFM as a new approach to detect inversion. Firstly, InvBFM uses Pindel, Delly, and Lumpy to generate inversion candidates. From the candidate inverting regions, the most significant features of inversions such as read pair orientation, one end unmapped and so on are assigned to specific values, and then the most effective 10 features are selected by combining chi-square test and experience. Finally, we use the SVM classifier to determine candidates as true inversions or not. All the real data in this paper comes from the 1000 Genomics Project. Because the inversions in real data are too few to train the classifiers, we use simulated data for model training and the real data for validation. The results show that our method is better than the existing three tools on recall and F1-score, ranks as second on precision, which is a little lower than Lumpy. In the future work, we will consider further mining the inversion features and exploiting the full use of real data to make inversion calling more effective.

## Methods

### Data

There are only a small number of validated inversions that have been released so far. The 1000 Genomes Project released a number of inversions. However, the number of called inversions from the 1000 Genomes Project is not very large. There are only 238 inversions recorded in Chromosome 11 in 102 samples. This is far from being enough to train the SVM classifier. Therefore, simulated data is used for training.

#### Simulated data

In this experiment, the simulated data is used as the training set of SVM classifier. The simulated data uses the reference genome (hs37d5.fa) from the 1000 Genomes Project. SimulateSeq [18] is used to simulated BAM files with different length, ISPE, and error rate on the reference genome. In order to avoid overfitting by the specific value of the parameters, we generated 13 sets of parameters in SimulateSeq, each parameter in every set is randomly taken within a range of values. For details, the range of inversion length is 500 to 6000, the ISPE is set in 300 to 500, and the error rate range is set from 0.003 to 0.005. There are also some parameters that affect the SimulateSeq less, we set the read length range from 70 to 150, the offset from 20 to 30, and the depth from 4 to 25. It is worth mentioned that the obvious limitation of SimulateSeq is that even if the error rate is introduced, the inversion area created is relatively clean, unlike the real inversion surrounded repetition or other SVs provided in 1000 Genomes Project. However, the results of cross-validation in the previous paper shown that the clean inversion can also train a better SVM classifier without overfitting, this is the reason why we choose the SimulateSeq. The 10 selected numerical features are extracted from these BAM files. Furthermore, these features from simulated bam are normalized with the candidate features of real bam, the specific approach is to employ scale function from preprocessing of sklearn. And then put scaled simulated features into the SVM classifier for training. This leads to a trained SVM classifier.

#### Real data

Real data from the 1000 Genomes Project is used as the test data. Inversions released by the 1000 Genomes Project are used as the benchmark in this paper. There are not many called inversions in the benchmark: the maximum number of inversions recorded in the benchmark for each sample on chromosome 11 is no more than four. There are totally 102 samples (BAM files) as the original data source. Chromosome 11 of the first 100 samples have more frequent inversions and chromosome 11 of the last two samples both have only one inversion according to the benchmark. The BAM files for these real data are low coverage, and they are released by the 1000 Genomes Project. The benchmark used in this paper is from the 8th version of vcf file updated in May 2017. A total of 238 inversions are reported on chromosome 11 of 102 samples. In addition, in order to verify the performance of InvBFM, we added another 102 samples of chromosome 11 (a total of 204 real data samples) also from 1000 Genomes Project to complete the cross-validation.

### High-level idea

InvBFM calls inversions by examining candidate inversions found by multiple inversion calling tools. The inversion calling is model-based. That is, InvBFM trains a classification model using SVM with various sequence-based features. In this paper, we use simulated data for model training. This is because there are only very limited real inversions available in the 1000 Genomes Project data release, and these real inversions are needed for validation. Our experience indicates that the model trained by the simulation data can still be useful when calling inversions in the real data.

### Workflow of InvBFM

**Fig. 2:**
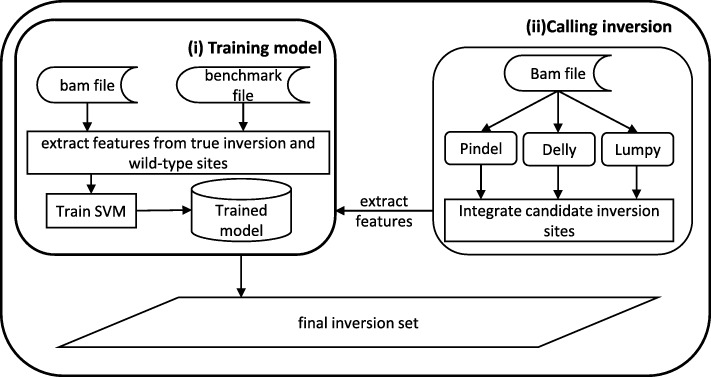
Workflow of InvBFM. It includes two major parts: (i) Training model. Bench-mark file is used to locate true inversion regions and non-SV regions, and then InvBFM extracts features from sequence reads around each label region to train a classification model by SVM. (ii) Calling inversion. Results of several tools are integrated as candidate inversion sites, then InvBFM extracts the same set of sequence features from sequence reads and calls inversion using the trained classification model

Our new method InvBFM takes mapped sequence reads on a given reference genome in the BAM format as input. There are two main parts for using InvBFM.as Fig.2 shown (i) Training model. InvBFM trains a classification model by SVM on simulated data. This model classifies a candidate inversion site to be either true inversions or wild-type from a set of collected sequence features. The sequence features are collected from the mapped reads near the inversion site. These features are informative about the presence of the inversion. (ii) Calling inversion. InvBFM extracts the same set of sequence features from sequence reads and calls inversion using the trained classification model. For calling, InvBFM runs multiple existing inversion calling tools, including Pindel, Delly and Lumpy. InvBFM then merges inversion calls from these tools to form the candidate inversion sites. InvBFM then calls inversions by examining each candidate site and classifying with the trained model. In order to improve accuracy, InvBFM mines the features of inversions and chooses a subset of more informative features in model training.

### Features

**Fig. 3:**
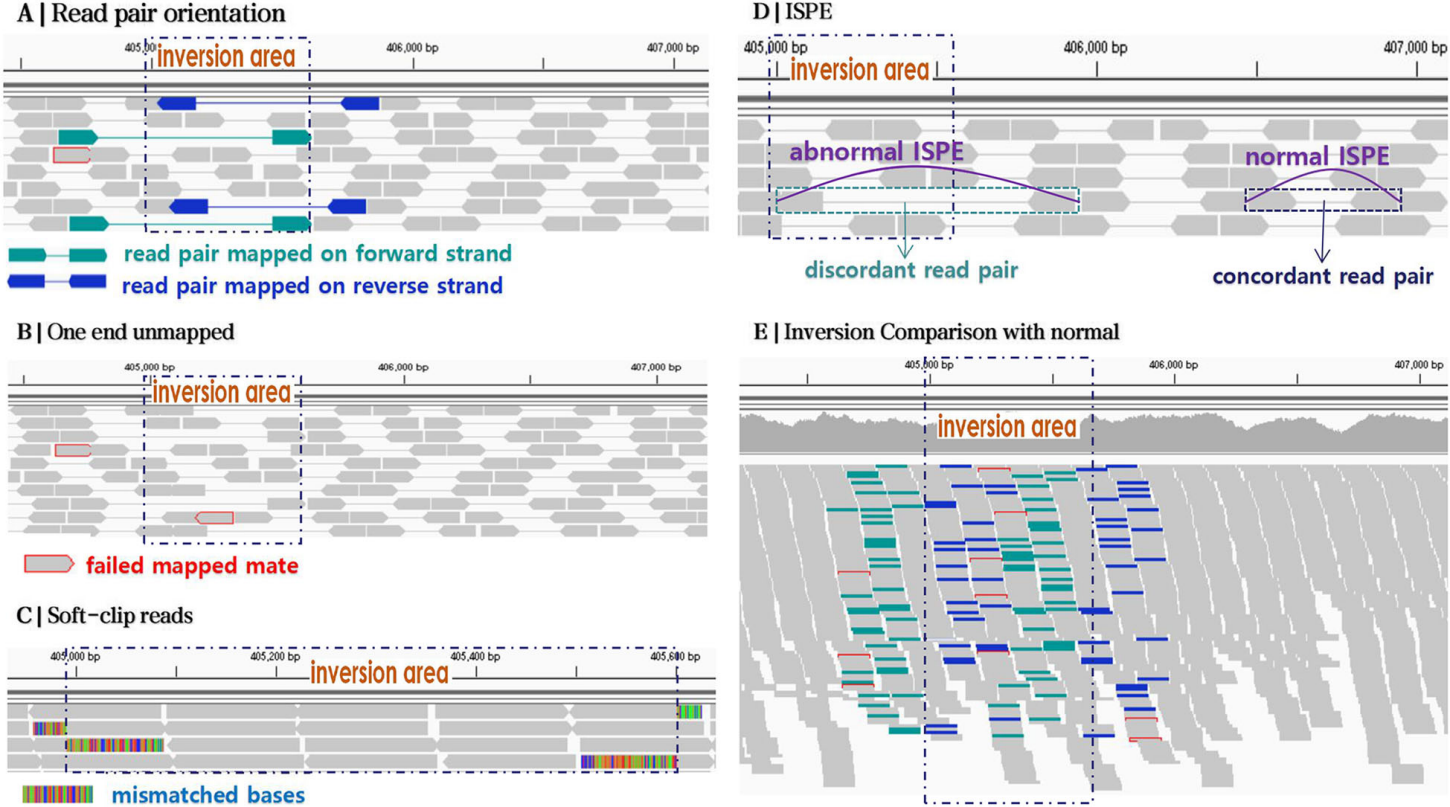
Inversion visualization in IGV. **a** Inversion region produces a large number of read pairs with the same orientation. The green read pair means both of the ends are mapped on the forward strand. The blue part means both of the ends are mapped on the reverse strand. **b** One end unmapped with the red line surrounded happens near the inversion, which means that an end mapping to the reference genome fails in the read pair near the inversion breakpoint. **c** When a read is mapped on a breakpoint of inversion, soft-clipped read occurs, in which the continuous bases are unmapped reflection as the color block. **d** ISPE is the distance between two ends in a read pair, ISPE is abnormally called discordant read pair perhaps indicates inversion occurs. **e** The overall effect of inversion on a genomic region

Compared with other genomic variants, inversion has some unique features. For example, read depth has been used as a main signature for calling deletions. Due to inversion is a balanced variation, read depth is not very informative. InvBFM uses some features, including the read pair orientation, one end unmapped, soft-clipped reads, concordance of ISPE and so on. Some of them are shown in Fig.3, which is produced by the Integrative Genomics Viewer (IGV) [19] on sequence data to visualize the features of inversions. It is important to note that we focus on diploid organisms in this paper. Therefore, an inversion may be presented in one or both copies of the chromosome. Also recall that we assume the sequence data have pairs (i.e. paired-end reads).

#### Read pair orientation

One of the most important features of inversion is that the two ends of a read pair has the same orientation while they mapped to the reference gene, which is different from the usual paired reads (where the two ends are oriented in the opposite direction). This happens when one end is outside the inversion and the other end is inside the inverted region. When reads are obtained from the paired-end sequencing technology, a read originates from the forward strand, and its mate originates from the reverse strand. In other words, two reads in a read pair usually are mapped on different strand. However, when inversion occurs, a read within the inversion area are mapped on the same strand as its mate. See Fig.3a for an illustration, the two reads linked by a straight line represent a read pair. The green reads linked by a straight line indicate that the reads in a read pair are both mapped to the forward strand of the reference and the blue indicates they are both mapped to the reverse strand. It can be observed that multiple reads mapped on the same strand are produced in the inversion region. Therefore, read orientation is an important feature of inversion.

#### One end unmapped

One end unmapped means that only one read of a read pair is successfully mapped to the reference. This occurs when one end of a read pair overlaps with the boundary of the inversion and becomes unmapped. As shown in Fig.3b, two reads are wrapped by a red line that are not linearly connected to another read. This means the read has a mate that is not mapped to the reference, the occurrence of inversions causes this abnormality. Within the inversion region, there are an increasing number of one end unmapped read pairs, which can be an important feature for inversion.

#### Soft-clipped reads

Soft-clipped read refers to a read partly mapped to the reference. Soft-clipped reads occur when one end of a read pair overlaps with the boundaries of the inversion region. Different from the one end unmapped case, the read is partially mapped (i.e. becomes a soft-clipped read). Thus, there tends to be more soft-clipped reads near the inversion boundary. This is shown in Fig.3c Color blocks indicate continuous bases that are mismatched with the reference. Each streak represents a mismatched base, and the soft-clipped read contains the color bar blocks.

#### ISPE

ISPE stands for the insert size of pair-end reads, indicating the distance between the two ends in a read pair. In a wild-type, ISPE usually has a range, which depends on the sequencing technologies. If the observed ISPE of mapped paired-end reads falls into this normal range, we say the read pair is concordant read pair. Otherwise, we say the read pair is discordant read pair. When one end of a read pair is outside the inversion area and the other end is inside the inversion area, the observed ISPE may be different from the true ISPE and the read becomes discordant. The concordant and discordant read pairs are shown in Fig.3d. This is more likely when the inversion is longer.

#### Other features

Besides the above features, other features can also be valuable for calling inversions, such as a read is uniquely or multiply mapped to the reference, whether perfectly mapped to reference, mapped quality and so on.

The features described above are specific features of inversions. Figure 3e shows inversion’s overall performance in IGV. It shows the case of inversion and also the case of wild-type. We can see the inversion features are enriched at the inversion site.

#### Feature expression

**Fig. 4:**
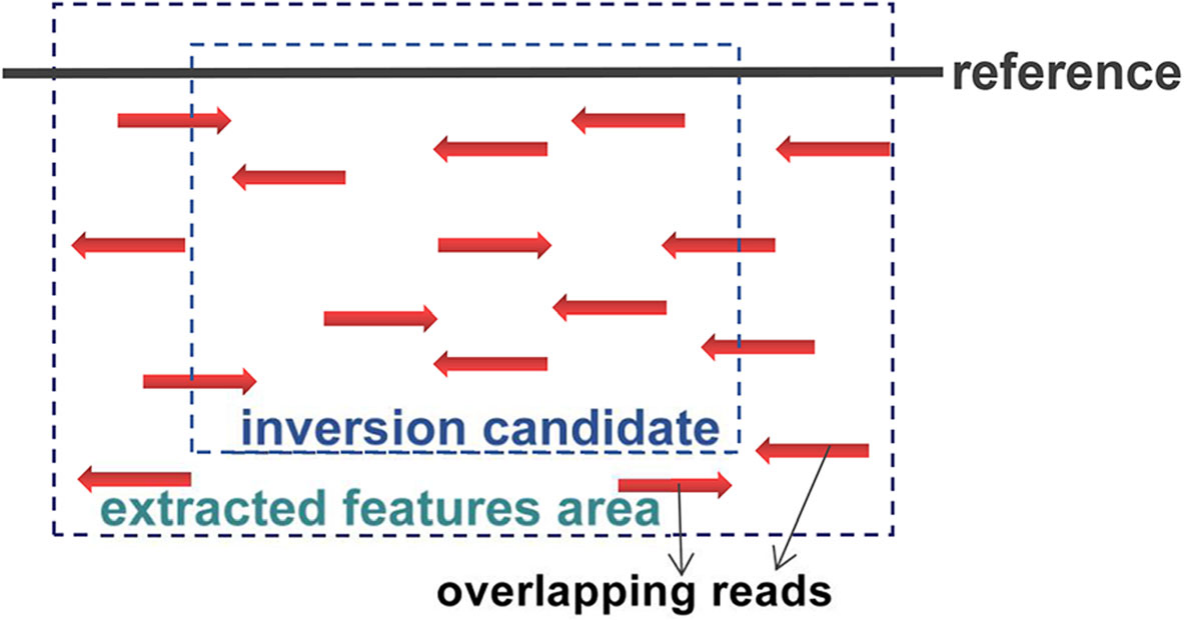
Extracted features in InvBFM. The range of extracted features is defined as the enlargement of ISPE by both left and right breakpoints of inversion area

InvBFM maps the features of inversion mentioned above into numerical features for subsequent processing. Table 8 shows more details of these features. The extraction of numerical features is based on the overlapping paired-end reads of the BAM file. Since some inversion features overlap with inversion breakpoints, the scope of the InvBFM fetch features is defined to be the left and right breakpoints of the ISPE of the paired-end reads, as shown in Fig.4. We use pysam [20] to extract features from the overlapping reads. Regarding the numerical features expressed in inversion, first, we extract the key information of overlapping reads. The information includes the XT value under the XT tag of read, which indicates the read is uniquely or multiply mapped to reference. Cigartuples indicate the specific bases’ mapped situations in reads. We mainly extract the number of bases of total mapped and soft-clip. Both of them contribute to the number of middle mapped quality and the number of clipped reads. We also look for its mate based on the read so that we can get the mapped direction of its mate. In addition, we also extract the length of the read, the mapped quality and its ISPE. The collection of the above information constitutes the set of read information required for the features, and the corresponding 15 numerical feature sets can be obtained by integrating the corresponding quantities of the information in the order of Table 8.

**Table 8:** Features. Each feature is assigned to an ID

Features	ID.	Description of numerical features
Uniquely/Multiply mapped	1	Number of uniquely mapped reads
	2	Number of multiply mapped reads
One end unmapped	3	Number of read pairs with one end unmapped
Soft-clip read	4	Number of clipped read
Mapped with error/error free	5	Number of mapped with error free
	6	Number of mapped with error
ISPE of read pair	7	Number of concordant pair whose ISPE is normal
	8	Number of discordant pair whose ISPE is abnormal
Mapping quality	9	Sum of mapped quality
	10	Number of low mapped quality
	11	Number of middle mapped quality
	12	Number of high mapped quality
Read pair orientation	13	Both mapped on reverse strand
	14	Both mapped on forward strand
	15	Both mapped on same strand

### Feature mining

Not all the features are equally informative about finding inversions. In order to mine the most efficient features, InvBFM performs feature selection on the initial extracted numerical features. InvBFM tests the correlation between each feature and its target value by using the chi-square test. The higher chi-square value is, the closer the relationship between the feature and the target is. When calculating the chi-squared value of a feature, we set O to be the observed frequency of features. E is the expected frequency of features. O is calculated from all the features and its output. E is calculated from the mean features and mean output. To estimate the difference between the observation frequency and the expected frequency, we use the chi-square test (3) to calculate the final chi-square value.

3 $$ {x}^2=\sum \frac{{\left(O-E\right)}^2}{E} $$

The highest 8 chi-square values obtained by the chi-square test are features 2, 4, 6, 8, 11, 13, 14 and 15 in Table 8. These 8 features are reserved as valid features. The reason why only using the 8 features with the highest chi-square value is that the feature whose chi-square value at the 9th indicates discordant pair. It is related to concordant pair which is already selected, and also selecting discordant pair as an effective feature causes feature redundancy. In addition, according to our experience, the number of one end unmapped and the sum of mapping quality are both important features for inversions. These are not included in the above 8 features selected by chi-square test. So, these two features are also added to the list of chosen features. The final feature set selected by InvBFM contains the following 10 features: 2, 3, 4, 6, 8, 9, 11, 13, 14, 15. The 10 features of InvBFM are more effective than the 8 features of chi-square test, which have been verified in previous results.

### Calling inversions

We first collect the called inversions as candidate inversions from existing tools, including Pindel, Delly and Lumpy. We use multiply tools here because existing tools for detecting inversion use different signatures: Pindel only uses split-mapped reads, and both Delly and Lumpy use ISPE of paired-end reads and split-mapped reads. This helps to find candidate inversions that are more likely to contain true inversions.

InvBFM uses the SVM classification to examine each candidate inversion to predict whether inversion occurs to get the final inversion set. Because there are a few validated inversions, the SVM classifier is trained with the simulated inversion. The SVM classifier then treats the generalization candidate inversions’ features from real data to get final inversion set.

In more details, InvBFM sets the number of simulated samples N_s_. Each sample has M_s1_ simulated inversions. So InvBFM extracts the number of N_s_*M_s1_ numerical features from the simulated inversions. These features from the inversions are set to 1. Similarly, features from the wild-type region are set to 0. The extracted features from the simulated data with the labels constitute the training data, which are scaled to train the SVM classifier. On the other hand, the numerical features of the 10 features are extracted according to the candidate inversion set of the real samples. These real data features are scaled and then put into the SVM classifier modeled by the simulated data to judge whether inversion occurs. The result of 1 indicates that InvBFM determines that the region is a true inversion, and 0 indicates that the region is a wild-type. Finally, InvBFM gets the final inversion set from the candidates with called label 1. The SVM classifier of InvBFM chooses a linear kernel with the penalty factor of 0.1 and the gamma of 20.

## Data Availability

All of the data mentioned to support our results in this paper are released by 1000 Genomes Project. For the detail, the reference genome in fa format is available at ftp://ftp.1000genomes.ebi.ac.uk/vol1/ftp/technical/reference/phase2_reference_assembly_sequence/hs37d5.fa.gz, the real data of BAM format file can be downloaded at ftp://ftp.1000genomes.ebi.ac.uk/vol1/ftp/phase3/data, the benchmark is available at ftp://ftp.1000genomes.ebi.ac.uk/vol1/ftp/phase3/integrated_sv_map/ALL.wgs.mergedSV.v8.20130502.svs.genotypes.vcf.gz.

## Abbreviations

IGV: Integrative Genomics Viewer
ISPE: Insert size from mapped paired-end reads
LumpyEP: Lumpy express
PCA: Principal component analysis
RBF: Radial basis function
SNPs: Single nucleotide polymorphisms
SVM: Support vector machine
SVs: Structural variations
